# New Facts about Antipirine

**Published:** 1885-02

**Authors:** 


TRANSLATIONS. *By* A. LAGORIO, M. D.

*New Facts About Antipirine.*—Rauk (*Bulletin Gén. de Theràp.*) has employed the new remedy, antipirine, hypodermically, to avoid the vomiting which is produced in some subjects when used internally, and from his observations he has drawn the following conclusions: 1. Antipirine is a prompt and a sure antipyretic in all febrile diseases, and especially in pneumonia, pleurisy, typhoid fever, acute rheumatism, erysipelas and tuberculosis; moreover, it does not produce any notable bad effects. 2. When used hypodermically, antipirine lowers the temperature more rapidly than when used internally. 3. To produce a lowering of the temperature by the hypodermic method, smaller doses are needed; two grams have been sufficient, while internally four to six grams must be given to have the same effect. 4. The best solution for the hypodermic use is that of 1 gram of antipirine in 50 centigr. of water. 5. This method does not produce any local nor general disturbances. 6. This method is always preferable to the way of the stomach (except in some cases, as in children or in feeble individuals), because smaller doses are used and the vomiting is avoided. 7. It seems that the remedy, antipirine, is destined to a great therapeutical future, its effects being sure and rapid, and the price not high.

The use of this remedy by other experimenters has always confirmed the results of Filehene, and Rauk-Biermer obtained splendid effects in several febrile diseases, (pneumonia, small-pox, erysipelas, etc.). Dr. Alexander gives great credit to this remedy in typhoid fever and phthisis (*Breslauer Arztli-zeitschrift*). Naunyn, of Koenigsberg, praises the good effects of antipirine in phthisis and in typhoid fever.

Generally the lowering of temperature commences after taking the first dose. Having reached the maximum of the lowering, this state will last generally five hours. The rise is made without any chill (kairine produces it). In no case any bad effect was noticed, although some patients took large quantities; one took 49 grams in eight days; another, 51 grams; a third, 15 grams in 24 hours. This shows that large doses can be taken without danger. The remedy has been impotent in the control of the paroxysms of intermittent fever.

Dr. Alexander, in a recent communication, referring to the new results of Biermer, at Breslau, is in some points in contradiction with Rauk. He is said to have been obliged to discontinue the hypodermic injections on account of the pain and abscesses produced by this process, while Rauk asserts that he has never had such accidents. To avoid these inconveniences, he administered the remedy in enemas: two table-spoonfuls of a solution ⅕ in 200 grams of water, repeated every hour, till a sufficient lowering is reached.

Prof. Riegel, of Giessen, to prevent the sweats frequently produced during the lowering of the temperature, gives two pills of agaricine of 0.005 gram. about a quarter of an hour before the first dose of antipirine. Atropine acts in the same way. (*B. Kl. Woch.*) Penzoldt and Sartorius have tried it in 21 children of different ages, in 18 cases of pneumonia, 1 of erysipelas, 1 of scarlet fever, and 1 of diphtheria.

In the beginning, as a precaution, the dose for children will be as many decigrams (less one or two) as the years of the little patient, viz.: five decigrams for a child six years old. They take the remedy willingly when mixed with syrup. The effect is also good when given in clysters.

